# America’s HIV Epidemic Analysis Dashboard: Protocol for a Data Resource to Support Ending the HIV Epidemic in the United States

**DOI:** 10.2196/33522

**Published:** 2022-02-10

**Authors:** Patrick Sean Sullivan, Cory R Woodyatt, Oskian Kouzouian, Kristen J Parrish, Jennifer Taussig, Chris Conlan, Harold Phillips

**Affiliations:** 1 Department of Epidemiology Rollins School of Public Health Emory University Atlanta, GA United States; 2 Oregon Health & Science University Portland, OR United States; 3 Office of Infectious Disease and HIV/AIDS Policy US Department of Health and Human Services Washington, DC United States; 4 Insignia Federal Group Falls Church, VA United States; 5 Conlan Scientific Bethesda, MD United States

**Keywords:** HIV, dashboard, data, data dashboard, infectious disease, infodemiology, surveillance, public health, United States, monitoring

## Abstract

**Background:**

The Ending the HIV Epidemic (EHE) plan aims to end the HIV epidemic in the United States by 2030. Having timely and accessible data to assess progress toward EHE goals at the local level is a critical resource to achieve this goal.

**Objective:**

The aim of this paper was to introduce America’s HIV Epidemic Analysis Dashboard (AHEAD), a data visualization tool that displays relevant data on the 6 HIV indicators provided by the Centers for Disease Control and Prevention. AHEAD can be used to monitor progress toward ending the HIV epidemic in local communities across the United States. Its objective is to make data available to stakeholders, which can be used to measure national and local progress toward 2025 and 2030 EHE goals and to help jurisdictions make local decisions that are grounded in high-quality data.

**Methods:**

AHEAD displays data from public health data systems (eg, surveillance systems and census data), organized around the 6 EHE indicators (HIV incidence, knowledge of HIV status, HIV diagnoses, linkage to HIV medical care, viral HIV suppression, and preexposure prophylaxis coverage). Data are displayed for each of the EHE priority areas (48 counties in Washington, District of Columbia, and San Juan, Puerto Rico) which accounted for more than 50% of all US HIV diagnoses in 2016 and 2017 and 7 primarily southern states with high rates of HIV in rural communities. AHEAD also displays data for the 43 remaining states for which data are available. Data features prioritize interactive data visualization tools that allow users to compare indicator data stratified by sex at birth, race or ethnicity, age, and transmission category within a jurisdiction (when available) or compare data on EHE indicators between jurisdictions.

**Results:**

AHEAD was launched on August 14, 2020. In the 11 months since its launch, the Dashboard has been visited 26,591 times by 17,600 unique users. About one-quarter of all users returned to the Dashboard at least once. On average, users engaged with 2.4 pages during their visit to the Dashboard, indicating that the average user goes beyond the informational landing page to engage with 1 or more pages of data and content. The most frequently visited content pages are the jurisdiction webpages.

**Conclusions:**

The Ending the HIV Epidemic plan is described as a “whole of society” effort. Societal public health initiatives require objective indicators and require that all societal stakeholders have transparent access to indicator data at the level of the health jurisdictions responsible for meeting the goals of the plan. Data transparency empowers local stakeholders to track movement toward EHE goals, identify areas with needs for improvement, and make data-informed adjustments to deploy the expertise and resources required to locally tailor and implement strategies to end the HIV epidemic in their jurisdiction.

## Introduction

The HIV epidemic remains a critical public health priority in the United States. Despite welcome advances in treatment, survival of people living with HIV [1], and HIV prevention [2], HIV remained the 9th leading cause of death among Americans aged 25-34 years in 2019 [3]. Realizing the full potential of the available powerful tools for HIV diagnosis, treatment and prevention will require coordination, bringing interventions to scale, and monitoring progress in HIV prevention, treatment, and care. To facilitate this process, in 2019, the US Department of Health and Human Services initiated the Ending the HIV Epidemic (EHE) initiative and operational plan, which provided a targeted infusion of new resources and support to local communities working together and with the federal government to end the HIV epidemic in America [4]. The goal of the EHE initiative is to accelerate HIV prevention progress and reduce the number of new HIV infections in the United States by 75% by 2025 and 90% by 2030 [4]. The initiative seeks to achieve this goal by providing those areas most in need with the additional expertise, technology, and resources required to scale up 4 key strategies (diagnose, treat, prevent, and respond) needed to end the HIV epidemic in their communities. To achieve maximum impact, EHE focuses its efforts in 48 priority counties, Washington, District of Columbia, and San Juan, Puerto Rico, where more than 50 percent of new HIV diagnoses occurred in 2016 and 2017, and an additional 7 priority states with a substantial rate of HIV diagnoses in rural areas, bringing the total number of priority jurisdictions to 57 [4].

Health statistics are recognized globally as critical to the development, implementation, monitoring, and evaluation of public health programs [5]. Accordingly, the EHE initiative requires the effective use of data to identify baseline levels of new HIV infections, establish 5-year and 10-year goals for reductions in transmissions, monitor interim progress toward these goals, and provide the jurisdictions responsible for public health with tools to easily illustrate progress and areas of opportunities to local prevention providers and stakeholders. Although existing online tools provide access to HIV surveillance and related data in graphical and tabular formats [6,7], the existing tools are not specific to the EHE initiative and are not organized around either the explicit goals of EHE, the EHE jurisdictions, or the 4 EHE key strategies. Further, experience with other dashboard tools suggests that the mechanisms by which dashboards can promote changes in public health programs include the opportunity for a comparative assessment of indicators among jurisdictions [8]. For these reasons, we developed and implemented a data visualization dashboard focused on the EHE jurisdictions, indicators, and program goals, which incorporates best practices from the existing tools. We then launched the dashboard and collected data on the usage of the dashboard during its first year of use. In this manuscript, we describe the rationale, stakeholder input process, and scope and sources of the data elements and the use of America’s HIV Epidemic Analysis Dashboard (AHEAD) in its first year of public availability.

## Methods

### Overview of AHEAD

AHEAD was launched on August 14, 2020, as a data visualization tool and primary source of tracking progress on the presidential initiative to end the HIV epidemic in America, the EHE. AHEAD was developed by the US Department of Health and Human Services through its Office of Infectious Disease and HIV/AIDS Policy in collaboration with the Office of the Assistant Secretary for Health, Centers for Disease Control and Prevention (CDC), Health Resources and Services Administration, Indian Health Service, National Institutes of Health, Substance Abuse and Mental Health Services, and Department of Housing and Urban Development.

**Figure 1:**
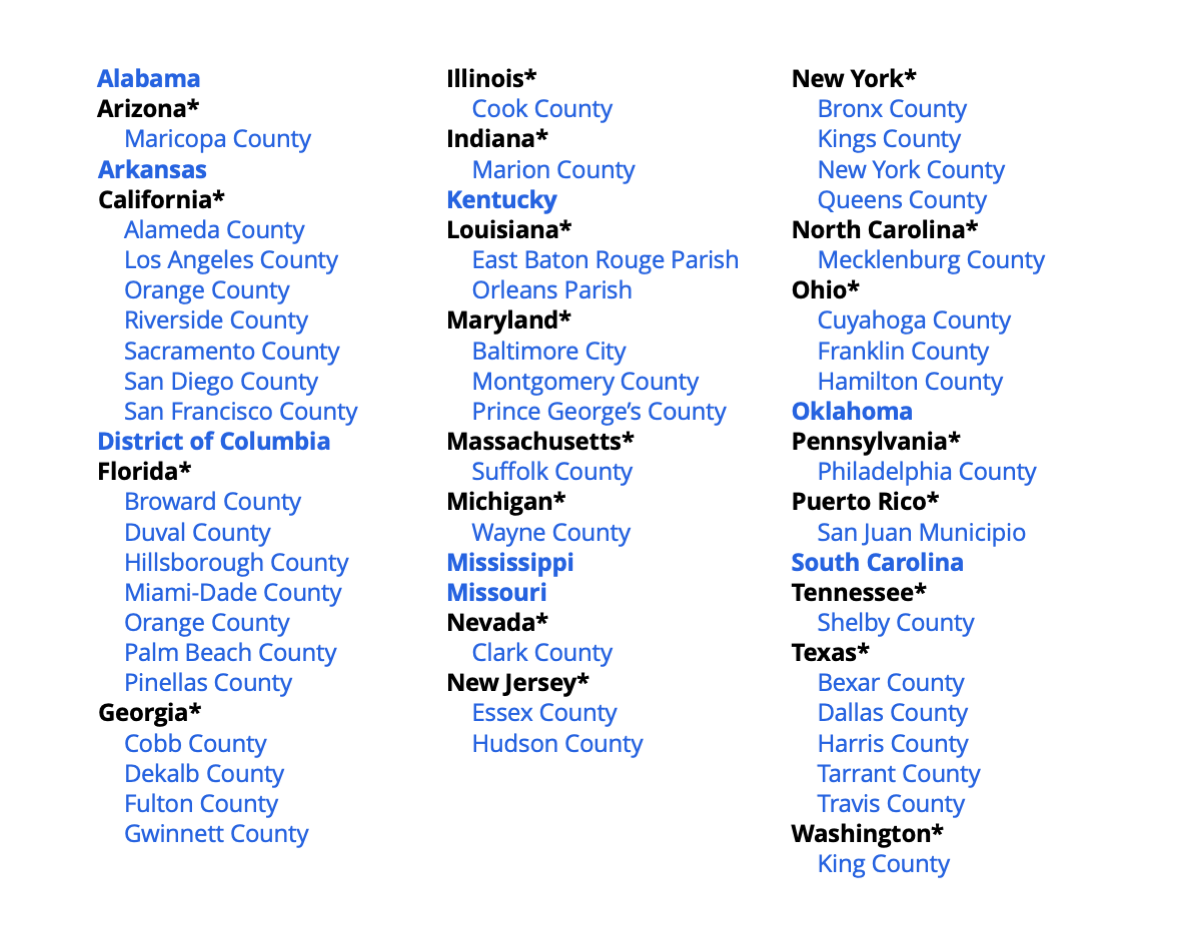
Ending the HIV Epidemic (EHE) priority areas. Asterisks indicate nonpriority states with one or more EHE priority counties.

AHEAD displays baseline and progress data for the nation and the 57 jurisdictions (Figure 1) for 6 indicators: HIV incidence, knowledge of HIV status, HIV diagnoses, linkage to HIV medical care, viral suppression, and pre-exposure prophylaxis (PrEP) coverage. AHEAD also displays indicator data for the remaining 43 US states, when available. Each indicator was chosen with specific public health goals in mind and in line with the 4 key strategies of the initiative: diagnose, prevent, treat, and respond [4]. Incidence measures the overarching goal of reducing new transmissions by 90% by 2030. Influencing just one indicator will not achieve the overall goals of the initiative; these indicators are contingent and progress in one will influence others. HIV diagnoses and knowledge of HIV status are both key to linking people to care and represent important steps on the HIV care continuum. Data have shown that, upon diagnosis, immediate linkage to care and treatment results in improved HIV outcomes [9], so it is important to track how these indicators change over time. Viral suppression and PrEP coverage will have the greatest impact on reducing new transmissions if they are scaled up and optimized for use among priority populations [10].

AHEAD displays both baseline (2017) and subsequent annual data for the 6 EHE indicators. 2017 was chosen as the baseline year to create the 2025 and 2030 goals because 2017 was the most recent year for which complete annual data were available when the initiative was announced and included stability of estimates for county-level incidence and knowledge of HIV status. Goals were calculated by applying programmatic goals (75% and 90% reductions in new HIV infections), as indicated by CDC data on HIV incidence. Goals are displayed for each of the Phase I EHE jurisdictions.

### Stakeholder Engagement

The development of electronic resources to promote public health requires input from diverse stakeholders, including data creators, intended users, representatives of public health organizations, and people living with HIV [8,11]. To guide the development of the content and functionality of AHEAD, we conducted a series of interviews with representatives of these groups to help identify the types of data and tailor the formats for display that would be most aligned with the needs of intended users. For all stakeholder interactions, online interview sessions were recorded with the permission of the participants, and field notes were recorded by dedicated notetakers during the discussion. Notes and review of recordings were used to develop summaries of individual stakeholder input and to identify recurring themes across stakeholder data.

#### Prelaunch Stakeholder Interviews

From July to August 2020, 13 interviews were conducted with diverse stakeholders from health departments and other organizations supporting public health and advocacy responses to the HIV epidemic. Stakeholders were selected without regard to their previous knowledge of the project. The participants were selected to represent multiple EHE jurisdictions in diverse geographic regions of the United States. The participants were provided with information about the purpose of AHEAD and planned content, and feedback was elicited regarding the overall project, including the features, functions, usability, and content value.

#### Limited User Assessment Interviews

Following the initial launch of AHEAD, we elicited further feedback from October and December 2020 via a series of limited user assessments. These were conducted to gather insight into stakeholder flow or types of use, intuitiveness, data scannability, usability of interactive functionalities, design aesthetics, topics for educational tools, strengths, weaknesses, opportunities, and possible threats. The feedback was used to update and refine the website further, ensuring the AHEAD project continued to be in tune and responsive to user needs.

### Data Sources

Of the 6 EHE indicators, 5 (HIV incidence, knowledge of HIV status, HIV diagnoses, linkage to care, and viral HIV suppression) reported on the site are from the National HIV Surveillance System (NHSS) [12]. NHSS is the primary source for monitoring HIV trends in the United States and CDC funds and assists state and local health departments to collect the information [13]. States and local jurisdictions, including the District of Columbia and Puerto Rico, have laws or regulations that require confidential reporting by name for adults and adolescents (ie, persons aged 13 and older) with a confirmed HIV diagnosis. After the removal of personally identifiable information, data from these reports are submitted to CDC. Health departments report these de-identified data to CDC so that information from around the country can be analyzed to determine who is being affected and why, which can inform where HIV prevention and treatment resources are most needed. The 6th indicator, PrEP coverage, uses 4 different data sources that are derived from population-based data sources and the published estimates of the size of groups with PrEP indications [14].

The demographic information used to create stratifications for data is based on information included in the NHSS records for people with diagnosed HIV infection [12]. For PrEP coverage, information about the age distribution and sex of PrEP users is derived from commercial pharmacy data sources using algorithms to identify likely PrEP users [15,16].

**Figure 2:**
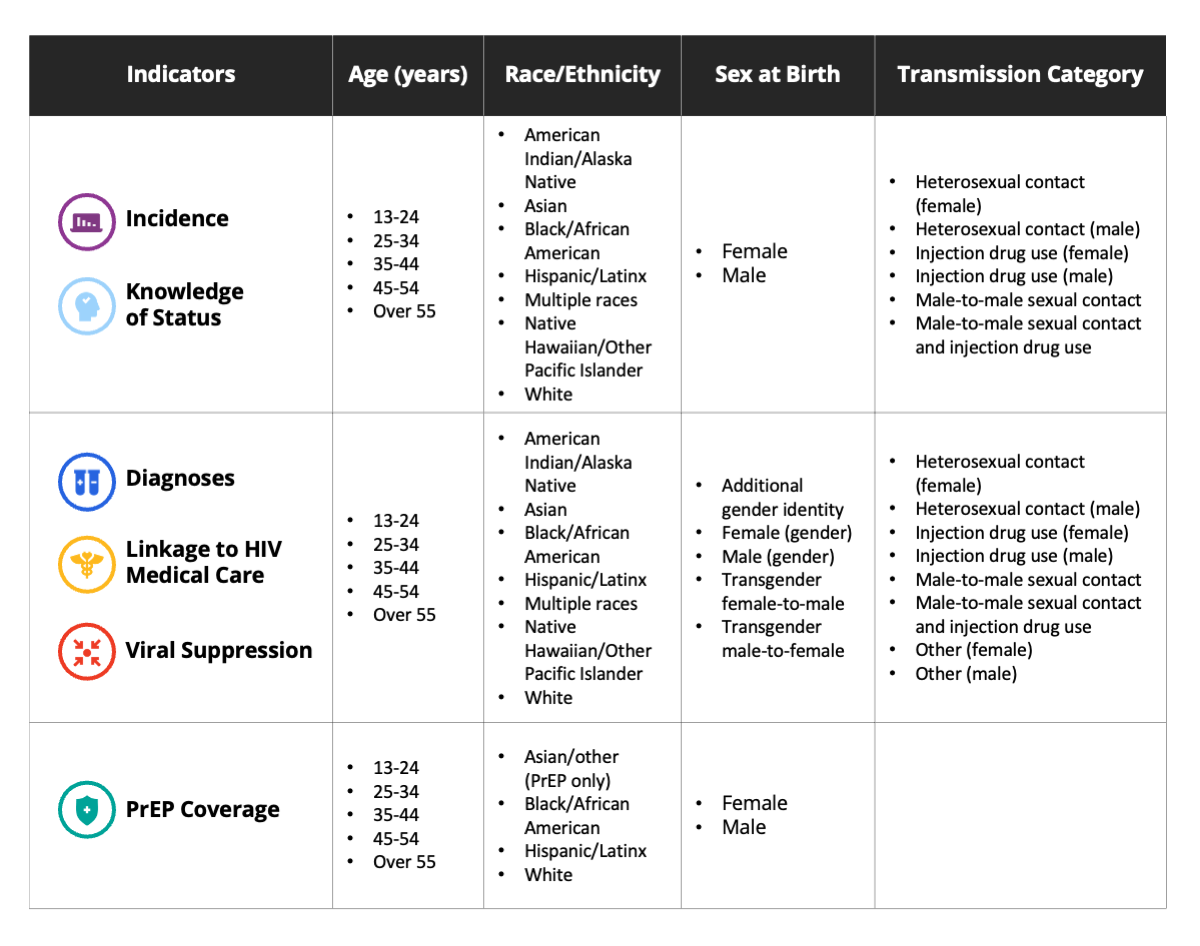
Ending the HIV Epidemic indicators and categories for demographic stratification for AHEAD (America’s HIV Epidemic Analysis Dashboard). PrEP: pre-exposure prophylaxis coverage.

The extent of stratification of indicators is based on the level of geography. At smaller geographic levels, stratification is more limited to prevent the possible indirect identification of individuals in small intersectional strata. Accordingly, stratification is more extensive at the national level on AHEAD; the indicators are presented overall, and stratified by age, race or ethnicity, sex at birth (for incidence, knowledge of HIV status and PrEP coverage) or gender (for diagnoses, linkage to HIV medical care, and HIV viral suppression), and transmission category. EHE goals are overall population goals, and demographic categories do not have separate 2025 or 2030 goals. The stratification criteria and categories are provided in detail in Multimedia Appendix 1 and are summarized in Figure 2.

### Available Data

**Figure 3:**
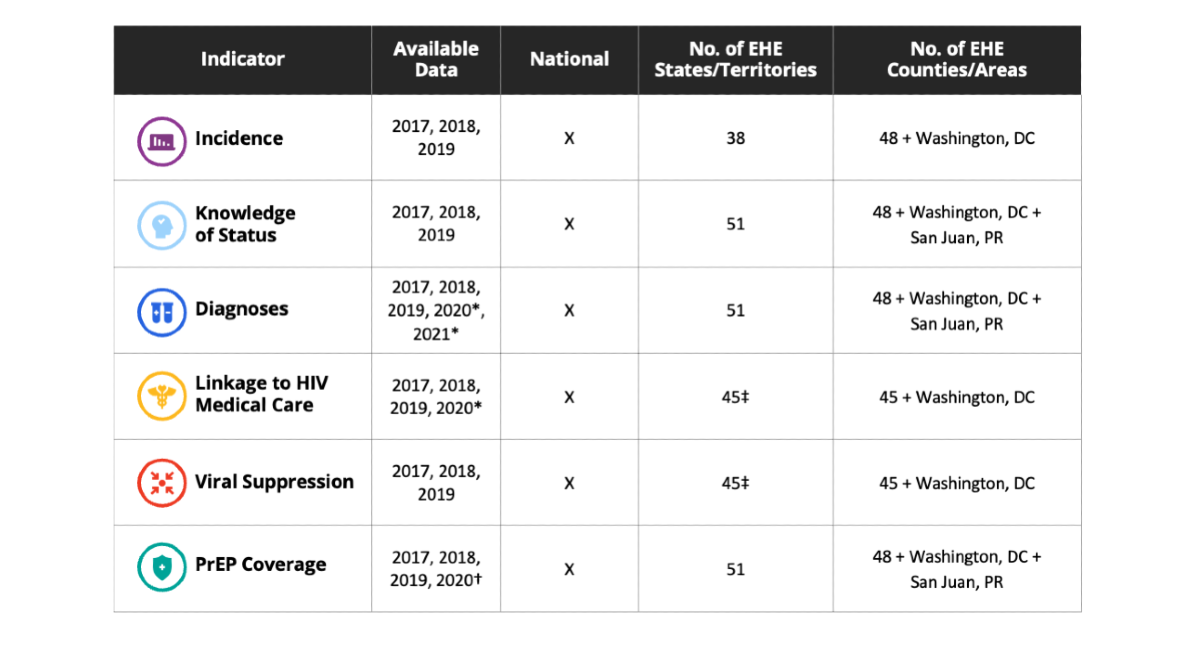
EHE indicators, available data as of Fall 2021, and the number of EHE states, territories, and counties with indicator data. EHE: Ending the HIV Epidemic; PrEP: pre-exposure prophylaxis coverage. *Data are preliminary as of March 2021; †Data are preliminary as of December 2020; ‡Data for years 2017 and 2018 are provided for 42 jurisdictions.

The data available through AHEAD (Figure 3) are organized around the EHE indicators [4]. Data are not available for all indicators, years, and jurisdictions for several reasons. For some indicators, data analyses are not initiated until sufficient time has passed to allow for substantially complete reporting of HIV-related laboratory results. Preliminary data are the earliest data released and are in essence a sneak preview to the complete annual data set. They do not have 12 months of reporting delay and can apply to both annual and cumulative quarterly data. Provisional data are data that do have at least 12 months of reporting delay and apply only to annual data.

### Functionalities for Data Display and Insights

Functionalities are provided in AHEAD to allow users to interact with the data in ways that promote a nuanced understanding of the data and suggest areas for possible programmatic focus, or for the collection of further contextual data to understand programmatic successes or needs for improvement. Depending on the levels of stratification available within the jurisdiction, such insights might allow the identification of specific subgroups (eg, Black people assigned female sex at birth) in which progress toward indicators is exemplary or limited compared to other subgroups. The illustration of goals allows users to assess whether the current progress toward indicators is sufficient to achieve eventual EHE goals, or whether the programs and efforts addressing specific indicators need to be modified or intensified to meet EHE goals.

Data can be viewed as either a chart or a table and by national, state, or county levels for each of the 6 indicators. Additional filters can be applied by age, race or ethnicity, sex or gender, and transmission category. Examples of data charts and tables are illustrated in the context of AHEAD tools for facilitating insights into the data.

#### Similar Jurisdiction Functionality

The “Show Similar Jurisdiction” functionality is based on a “nearest neighbor” data analysis; in this case, distance is not geographical distance, but rather based on a distance matrix computer science algorithm [17], which identifies the 3 most similar EHE counties or areas based on a given jurisdiction’s EHE indicator data points. The purpose of using a nearest neighbor analysis is to examine progress across all 6 indicators and identify other jurisdictions that are quantitatively most similar to the selected jurisdiction. These similar jurisdictions may be interested in communicating with and exploring similar strategies to better achieve EHE goals.

#### Implementing EHE Goals at the Jurisdictional Level

**Figure 4:**
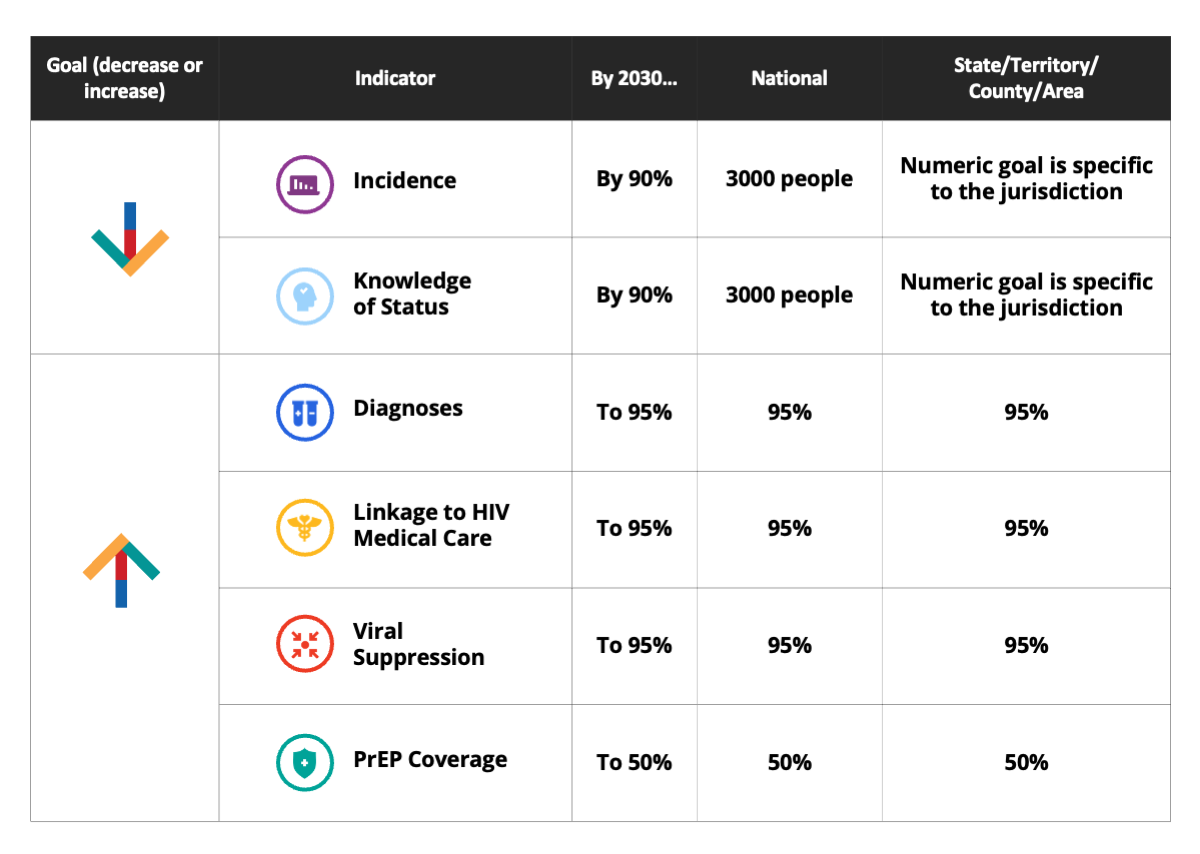
Ending the HIV Epidemic goals and indicators at the national and jurisdictional levels, United States, through 2030.

Although EHE goals are overall national goals [4], achieving national goals will not be possible unless most or all of the EHE jurisdictions meet these same goals at the local or state level. The focus on jurisdictional goals is also important from the perspective of health equity [18]. Therefore, it is useful to apply the EHE goals to the jurisdictional level. For HIV incidence, the jurisdiction-specific goals are derived based on the baseline levels of incidence in the jurisdiction and are therefore specific to each jurisdiction. Goals around care continuum indicators are proportional in both national and subnational jurisdictions (Figure 4).

**Figure 5:**
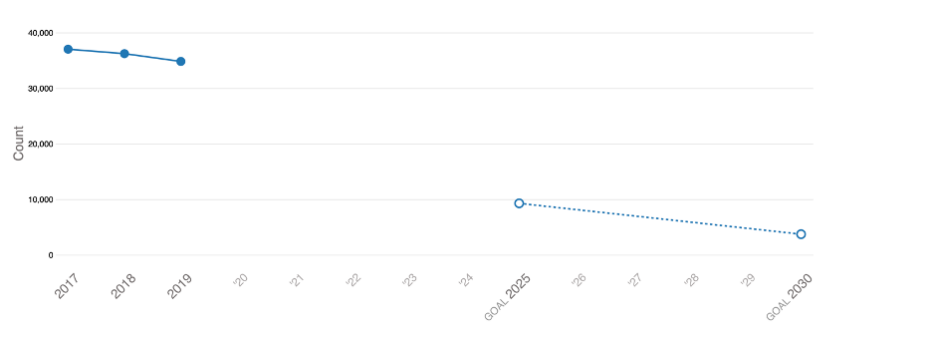
HIV incidence by year, United States, 2017-2019, and Ending the HIV Epidemic goals for 2025 and 2030.

**Figure 6:**
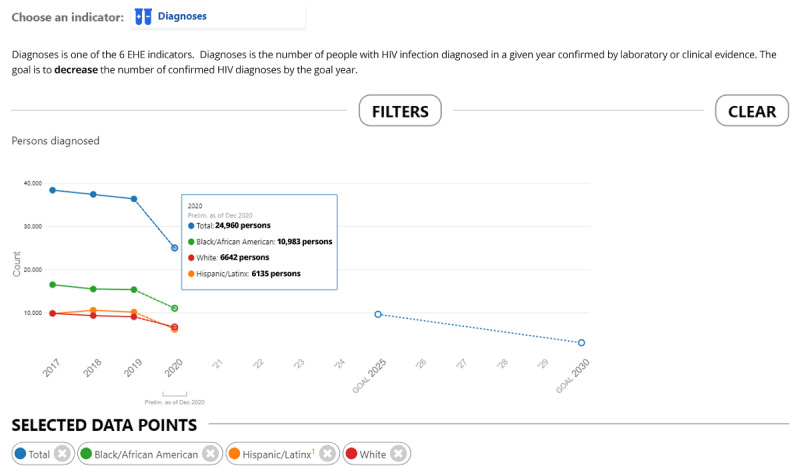
HIV Diagnoses overall and among Black, White, and Hispanic people, by year of diagnosis, United States, 2017-2020, and 2025 and 2030 EHE goals; the 2020 data displayed in this figure are preliminary as of December 2020. Interpret preliminary 2020 data with caution as the impact of COVID-19 on 2020 data year is not known to date. EHE: Ending the HIV Epidemic.

**Figure 7:**
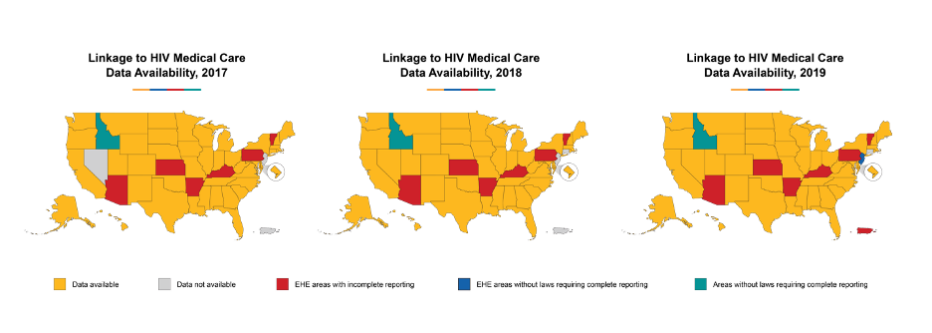
Linkage to HIV medical care data availability, 2017-2019. EHE: Ending the HIV Epidemic.

The methods for deriving individual indicators from national data sources have been described previously, and our implementation of those methods for AHEAD are summarized in Multimedia Appendix 2. AHEAD adds value to the existing data sources by illustrating the annual trends for each indicator by jurisdiction and by displaying the historical trends in relation to the 2025 and 2030 goals (Figure 5). At some geographic levels and for some indicators, stratified data are available to show progress in important subgroups (eg, groups by race or ethnicity; Figure 6). In some cases, not all indicators are available in all jurisdictions (Figure 7).

#### Downloadable Materials

Data are available for download in the following 3 formats: active data (CSV), all data (Excel spreadsheet), or by chart (PNG). Additional materials include a 1-page document outlining the features of AHEAD, an AHEAD user guide, as well as a comprehensive toolkit for jurisdictions, community-based organizations, advocacy organizations, and federal agencies (including sample tweets, Facebook posts, Instagram posts, e-blasts, and newsletter blurbs).

#### Infographics

**Figure 8:**
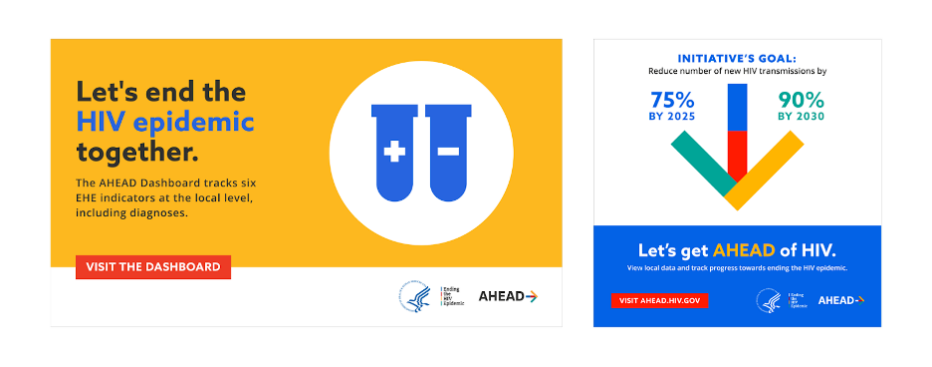
Examples of AHEAD infographics optimized for social media dissemination. AHEAD: America’s HIV Epidemic Analysis Dashboard; EHE: Ending the HIV Epidemic.

Infographics (Figure 8) are graphical panels that can be designed to illustrate key messages of the EHE plan, emphasize EHE goals, or illustrate specific data elements in ways that might increase access to or comprehension of information. Infographics for AHEAD are optimized for dissemination through social media channels and are freely available for download on the site.

#### Other Resources

AHEAD provides users access to other valuable sites and resources including HIV.gov’s site Ready, Set, PrEP [19], notice of funding opportunity announcements, and a locator tool that provides geo-located HIV testing, PrEP, and care services sites. In addition, blog posts are prepared to provide information about how to use and interpret the data on AHEAD. To date, HIV.gov has released the following series of blog posts related to AHEAD:

1. AHEAD Dashboard - EHE Indicators: PrEP Coverage
2. AHEAD Dashboard and Stakeholders
3. AHEAD Dashboard Enhances Functionality
4. AHEAD Dashboard Updated to Reflect 2020 Data on Two Key Indicators
5. AHEAD Dashboard: Launched
6. AHEAD Dashboard: Understanding the Sources of EHE Indicator Data
7. AHEAD Informational Webinar Materials Available
8. AHEAD: Monitoring Progress Toward Achieving the Nation’s Viral Suppression Goals
9. AHEAD: Tracking Linkage to HIV Medical Care Data to End the HIV Epidemic
10. Coming Soon: The AHEAD Dashboard
11. Ending the HIV Epidemic with Data: Why AHEAD Data Matters
12. Ending the HIV Epidemic: 6 Indicators. 4 Strategies. 1 Goal - The AHEAD Dashboard
13. HOPWA Resource Tool Helps Jurisdictions Plan for and Evaluate Housing Needs
14. New HIV Indicator Data on AHEAD
15. Sign-Up for Free TA on AHEAD
16. The Impact of Incidence Data on Ending the HIV Epidemic
17. What You Need to Know About the 6 EHE Indicators: The AHEAD Dashboard

## Results

### Website Sessions and Users

**Figure 9:**
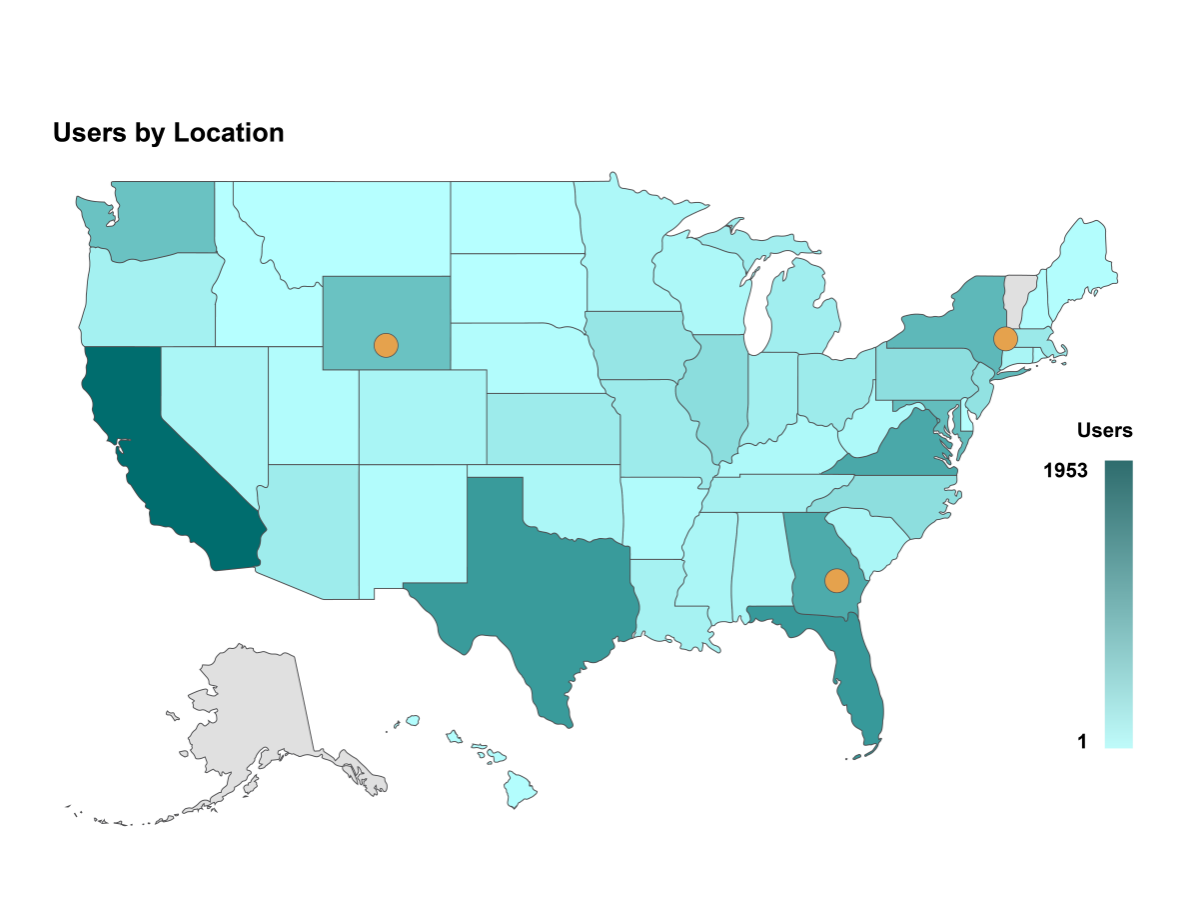
AHEAD (America’s HIV Epidemic Analysis Dashboard) users by location and number of users per jurisdiction, August 2020 to July 2021.

From August 2020 to July 2021, on average, AHEAD garnered approximately 2646 sessions per month. In total, AHEAD has received 29,110 sessions since August 2020. The average session duration was 2 minutes and 18 seconds, and users are engaging with 2.35 pages per session, indicating the AHEAD audience is engaging with the content. During the same period, there have been approximately 1013 downloads from the AHEAD site of the materials available to users indicating AHEAD’s audience is using the resources provided on the site. There were 6932 (23.81%) returning visitors; thus, about one-quarter of the users visited the site more than once. The top locations that are being selected within the data page are Alabama, California, Florida, Georgia, and Alabama. The states with the highest rates of engagement are California, Florida, and Georgia (Figure 9). By channel, most sessions (11,120/29,110, 38.2%) were referred from Google/organic. Moreover, 9636 (33.1%) out of 29,110 sessions were referred from Bing/organic; 5938 (20.4%) out of 29,110 sessions were referred from other search engines; and 2416 (8.3%) out of 29,110 sessions were accessed directly through the site URL.

### Uses by Media

AHEAD can also be used by media channels as a resource to increase the awareness of the HIV epidemic and the EHE plan for America. For example, in July 2021, Edge Media Network reported on AHEAD as a new site that tackles HIV at the local level [20]. A local network news affiliate used data from the site to support a study about the HIV epidemic in Florida [21]. Additional uses of the data include a story by a national online news site focusing on LGBT (lesbian, gay, bisexual, and transgender) health, which highlighted the more granular county-level data on AHEAD and described the site as an important new tool to tackle the epidemic at a local level.

## Discussion

Ambitious public health programs are unlikely to be successful without transparent, up-to-date, and accessible tools for monitoring public progress at each jurisdictional level responsible for public health response. In the case of the EHE plan, national goals for ending the epidemic will be difficult or impossible to achieve unless there is substantial progress toward the goals in each of the 57 EHE jurisdictions. For these reasons, AHEAD was created to supplement existing data visualization tools to (1) provide focus on the EHE jurisdictions; (2) tailor data elements to align with EHE indicators; (3) allow visualization of 2025 and 2030 goals along with data on progress of individual jurisdictions; and (4) allow jurisdictions to see their progress and to compare their progress to peer jurisdictions. AHEAD contextualizes where we currently stand, the progress being made, and how close we are to achieving our goals.

A previous analysis of the uses of data from an HIV dashboard [7] suggested that the examination of local data can lead directly to public health responses that are informed by the geographic areas or populations at highest risks for poor outcomes [8]. Prior uses of comparative geographic data on morbidity and service locations have led to the development of programs that geographically focus on new resources for prevention or care services [8]. Thus, we propose 3 ways that jurisdictions and stakeholders can use AHEAD to inform their local planning and advocacy to drive toward meeting EHE goals: evaluation of progress toward EHE goals, examination of stratified analyses to identify subgroups of people who might need enhanced services, and comparison with peer jurisdictions to promote tailored peer-to-peer sharing of best practices and the development of peer networks.

Ideally, jurisdictions will use the data and features in AHEAD to evaluate their progress periodically and assess whether their pace of progress is sufficient to meet 2025 and 2030 goals. Even in the absence of more formal analysis or statistical inference, the inspection of Figure 5 and extrapolation of the trends in HIV incidence from 2017-2019 suggest that the current pace of reductions in incidence will not lead to sufficient reductions to meet 2025 or 2030 goals. The examination of the continuum indicators might provide insight into more specific interim goals and priority populations for programmatic intervention.

Second, AHEAD provides online tools to evaluate progress toward individual EHE goals in stratified groups of people. For example, a visualization of PrEP coverage for persons with a PrEP indication (Multimedia Appendix 3) indicates that declines in PrEP coverage overall in 2020 compared to 2019 were larger for 16- to 24-year-olds than for older persons. This observation should lead to hypotheses about why PrEP coverage differentially decreased in 2020 for younger people (eg, COVID-19 impacts and lower autonomy in accessing health care providers) and consideration of programs to facilitate access to young people who might benefit from PrEP.

Third, AHEAD offers the opportunity for local public health officials to compare their jurisdiction to other similarly positioned jurisdictions. These comparisons can be made based on prior knowledge of jurisdictions with similar demographics or policy settings or can be identified empirically using built-in tools to identify the underlying similarities in factors that might shape risks and effectiveness of public health interventions. This allows for areas to identify “peer” jurisdictions, with whom these peers can discuss challenges, and what solutions have been effective for them. For example, a jurisdiction experiencing slow progress in a particular indicator could look for another county or area that has been successful in that indicator and contact that jurisdiction to brainstorm possible solutions together.

The data used in AHEAD are all population-based, and the underlying HIV surveillance system [12] is routinely evaluated according to standard criteria for completeness, timeliness, and other key performance metrics [22]. However, surveillance data on HIV diagnoses are likely minimal counts of people with HIV, because not everyone who acquires HIV is tested and diagnosed, and some states allow anonymous testing. People tested for HIV anonymously will not be reflected in the NHSS data until confidential confirmatory testing is completed and the information reported to CDC. Similarly, PrEP use data that are developed using a commercial pharmacy data source, which comprises about 80% to 92% of US prescriptions filled in a commercial pharmacy; however, these data are subject to bias if the patrons of included pharmacies differ from those of excluded pharmacies [16,23]. Finally, users should be aware that the data displayed on AHEAD for specific jurisdictions might not be the same as the data reported in local surveillance reports. This is because CDC deduplicates case reports when one individual is reported by multiple jurisdictions [24], and because CDC data are statistically adjusted to assign transmission categories to people reported with missing risks for HIV acquisition [25].

The initial version of AHEAD displays baseline data for the nation and jurisdictions (where data are available) for each of the 6 indicators. In 2021, additional features and information about the EHE initiative will be added to the site. As AHEAD evolves, the site will become more interactive, integrate more data sources, and provide both national and jurisdictional leaders with the tools to make decisions on resources and interventions in their communities. This site will also serve as a primary site to share information about the EHE progress, interventions, and success stories from the field.

Surveillance data are meant to drive public health action [26]. HIV surveillance data have been described as “the conscience of the HIV epidemic” [27]. As we look to the initial assessment of EHE goals in 2025, we must develop, deploy, and promote tools that help make high-quality data accessible to stakeholders in all aspects of ending the HIV epidemic—providers, public health professionals, community organizations, policymakers, researchers, academics, and advocates. AHEAD occupies a unique space among online HIV data tools in its focus on the EHE jurisdictions and its benchmarking to EHE indicators and initiative goals. Data visualization tools democratize data [28], and increasing the number of people interacting with data on the HIV epidemic can only enhance our efforts toward ending the epidemic.

## Appendix

Multimedia Appendix 1 Details on demographic groups used for stratification in AHEAD (America’s HIV Epidemic Analysis Dashboard).

Multimedia Appendix 2 Methods for calculation of indicators.

Multimedia Appendix 3 Stratified analysis of PrEP (pre-exposure prophylaxis coverage) illustrating the importance of stratified data visualization.

## Abbreviations

AHEAD: America’s HIV Epidemic Analysis Dashboard
CDC: Centers for Disease Control and Prevention
EHE: Ending the HIV Epidemic
LGBT: lesbian, gay, bisexual, and transgender
NHSS: National HIV Surveillance System
PrEP: pre-exposure prophylaxis coverage
