# Supplementary material for: America’s HIV Epidemic Analysis Dashboard: Protocol for a Data Resource to Support Ending the HIV Epidemic in the United States
Source: JMIR Public Health Surveill. 2022 Feb 10;8(2):e33522. doi: 10.2196/33522 (PMC8874801; doi:10.2196/33522)
Supplement: Multimedia Appendix 1 [file publichealth_v8i2e33522_app1.docx]

*Age Groups*

The data shown are only for adults and adolescents (i.e., persons aged 13 years and older for five indicators, and 16 and older for PrEP coverage). For diagnosis data, age is based on the person’s age at HIV diagnosis. For data on persons living with diagnosed HIV infection, the age is based on the person’s age as of December 31 of the queried year. Ten-year age groups are used with HIV surveillance data to ensure data security and confidentiality.

*Race/Ethnicity*

Data are grouped into the following racial/ethnic categories (each race category is non-Hispanic): American Indian or Alaska Native, Asian, Black or African American, Hispanic or Latinx, Native Hawaiian or other Pacific Islander, White, and Multiracial. Persons of Hispanic/Latinx ethnicity can be of any race. Some HIV prevalence cases are missing information on the individual’s race/ethnicity. These unknown racial/ethnic groups are not displayed. Therefore, if you summarize the data by race/ethnicity, they may not match the state and national totals overall.

PrEP data has different race/ethnicity categories due to the source of the data. The categories for PrEP are: Black/African American, Hispanic/Latinx, Other, and White.

*Sex and Gender*

Sex designations (male and female) are based on a person’s sex assigned at birth. National-level data for the following indicators are stratified by sex (because information on gender is not available): incidence, knowledge of status, and PrEP coverage.

The term “gender” in this dashboard refers to gender identity – a person’s internal understanding of their gender, or the gender with which a person identifies – and includes the following categories: male, female, transgender male-to-female, transgender female-to-male, and other gender (e.g., bigender, gender queer, two-spirit). National-level data for the following indicators are stratified by gender: diagnoses, linkage to HIV medical care, and viral suppression.

Transgender is an umbrella term that is used to identify persons whose sex assigned at birth does not match current gender identity or expression. Information on gender identity is still not consistently collected or documented in the data sources used by HIV surveillance reporting jurisdictions. Thus, HIV data by gender remain limited. HIV surveillance personnel collect data on gender identity, when available, from sources such as case report forms submitted by health care or HIV testing providers and medical records, or by matching with other health department databases (e.g., Ryan White program data).

*Transmission Categories*

Transmission categories is the term for the classification of cases that summarizes a person’s possible HIV risk factors; the summary classification results from selecting, from the presumed hierarchical order of probability, the one risk factor most likely to have resulted in new HIV infections. The exception is men who had sexual contact with other men and also injected drugs; this group makes up a separate transmission category.

Persons whose transmission category is classified as male-to-male sexual contact include men who report sexual contact with other men (i.e., homosexual contact) and men who report sexual contact with both men and women (i.e., bisexual contact). Persons whose transmission category is classified as injection drug use (IDU) are persons who injected non-prescribed drugs. Persons whose transmission category is classified as heterosexual contact are persons who have ever had specific heterosexual contact with a person known to have, or to be at high risk for, HIV infection (e.g., a person who injects drugs). All other transmission categories have been collapsed into “Other.” The “Other” transmission category includes: hemophilia, blood transfusion, perinatal exposure, and risk factor not reported or not identified.

Due to the large number of cases reported without transmission category information, transmission category data are statistically adjusted using multiple imputation techniques to account for missing transmission category information in cases reported to CDC. Because of this statistical adjustment, the values across individual transmission categories may not sum to the national total for incidence, knowledge of HIV status, linkage to HIV care, diagnoses, and viral suppression.
