# Supplementary material for: America’s HIV Epidemic Analysis Dashboard: Protocol for a Data Resource to Support Ending the HIV Epidemic in the United States
Source: JMIR Public Health Surveill. 2022 Feb 10;8(2):e33522. doi: 10.2196/33522 (PMC8874801; doi:10.2196/33522)
Supplement: Multimedia Appendix 2 [file publichealth_v8i2e33522_app2.docx]

*Indicator: HIV Incidence*

Estimates of annual HIV infections (incidence) are based on NHSS data for persons aged ≥ 13 years.[[28]](https://paperpile.com/c/nU1tvG/XpJF) Incidence measures the number of all new infections, whether diagnosed or undiagnosed, in a given year. Incidence estimates are useful for planning and for allocating funds, as well as evaluating the impact of prevention programs. For example, these estimates can be used to assess changes in characteristics of persons most at risk for acquiring HIV infection.

HIV incidence estimates were produced using the result of the first CD4+ T-lymphocyte [CD4] test after HIV diagnosis and an estimation method based on a CD4 depletion model (referred to hereafter as the “CD4 model”). The first CD4 test results after HIV diagnosis are routinely collected by all jurisdictions as part of the NHSS. Assuming that no treatment has been received, the CD4 cell count can be used to estimate the time from infection to the date of the CD4 test. The CD4 model was applied to NHSS data to estimate the distribution of delay from infection to diagnosis and then to produce estimates of HIV incidence and prevalence among adults and adolescents. The use of the CD4 model is possible because of improvements in HIV case surveillance. Reporting of the first CD4 test result after diagnosis of HIV infection is a required data element on the HIV case report form; however, completeness of reporting varies among states and local jurisdictions. Estimates should be interpreted with caution for EHE areas (including Phase 1 EHE states, EHE jurisdictions, or states that contain EHE jurisdictions) that do not have laws requiring complete reporting of laboratory data or have incomplete reporting.

The estimates of the incidence of HIV infection (diagnosed and undiagnosed) among adults and adolescents were obtained in the following steps:

Among all HIV diagnoses reported by states to the CDC, the date of HIV infection was estimated for each person with a CD4 test by using a CD4 depletion model. The distribution of delay (from HIV infection to diagnosis) was used to estimate the annual number of HIV infections (new infections in a given year), which includes persons with diagnosed infections and persons with undiagnosed infection. To reflect model uncertainty, all estimates were rounded to the nearest 100 for estimates of more than 1,000 and to the nearest 10 for estimates of less than 1,000.

The relative standard error (RSE) is a measure that shows how large the standard error is, relative to the size of the estimated value. It is calculated by dividing the standard error of an estimated value by the estimated value itself, and then multiplied by 100 and expressed as a percent. Smaller RSEs are indicative of more reliable results, and larger RSEs indicative of less reliable results. Estimates with a relative standard error (RSE) of ≥30% do not meet the standard of reliability and are represented in the following way: Estimates with an RSE of 30% - 50% are marked with an asterisk (*), indicating that they should be used with caution. Estimates with an RSE>50 percent are not shown, and are replaced with the phrase “Data N/A due to high relative standard error.”

*Indicator: Knowledge of HIV Status*

Knowledge of HIV status is estimated as the percentage of persons with HIV who have received a diagnosis. Estimates are derived by using HIV surveillance data and CD4 data for persons aged ≥13 years at diagnosis. HIV prevalence (the number of persons living with diagnosed or undiagnosed infection) is the denominator for this indicator and is estimated by adding the number of persons with undiagnosed HIV infection [derived from the estimated annual number of HIV infections as described above in the section “Estimated HIV incidence (diagnosed and undiagnosed)”] to the number of persons living with diagnosed HIV infection (reported to NHSS).[[28]](https://paperpile.com/c/nU1tvG/XpJF) Knowledge of HIV status (also known as percentage diagnosed or percentage aware) is determined by dividing the number of persons with diagnosed HIV by the total HIV prevalence for each year. The number of persons with diagnosed HIV is based on HIV surveillance data reported to CDC from 50 states and the District of Columbia for persons aged ≥ 13 years.

Completeness of reporting varies among states and local jurisdictions. Estimates should be interpreted with caution for EHE areas (including Phase 1 EHE states, EHE jurisdictions, or states that contain EHE jurisdictions) that do not have laws requiring complete reporting of laboratory data or have incomplete reporting.

The relative standard error RSE is a measure that shows how large the standard error is, relative to the size of the estimated value. It is calculated by dividing the standard error of an estimated value by the estimated value itself, and then multiplied by 100 and expressed as a %. Smaller RSEs are indicative of more reliable results, and larger RSEs indicative of less reliable results.

Estimates with a relative standard error (RSE) of ≥30% do not meet the standard of reliability and are represented in the following way: Estimates with an RSE of 30% - 50% are marked with an asterisk (*), indicating that they should be used with caution. Estimates with an RSE>50 percent are not shown, and are replaced with the phrase “Data N/A due to high relative standard error.” To reflect model uncertainty, all estimates were rounded to the nearest 100 for estimates of more than 1,000 and to the nearest 10 for estimates of less than 1,000.

Please note that at the time of development of this dashboard, the estimates on knowledge of HIV status for the year 2018 were considered preliminary, as they were based on deaths that have been reported only as of December 2019.

*Indicator: HIV Diagnoses*

For the baseline value of the HIV diagnoses indicator, data on diagnosed HIV infection include persons aged ≥13 years with laboratory-confirmatory evidence of HIV infection during January 1, 2017 through December 31, 2017 and reported to CDC through December 31, 2019. [[29]](https://paperpile.com/c/nU1tvG/LJ3A) Data are based on residence at time of diagnosis of HIV infection. Preliminary 2020 diagnoses data represent the number of reported HIV infections among persons aged ≥13 years that were confirmed through laboratory or clinical evidence during a calendar year. Data presented by quarter are preliminary and include cumulative counts of HIV diagnoses through the specified quarter of the calendar year. Data presented by quarter are preliminary.

*Indicator: Linkage to HIV Medical Care*

The linkage to HIV medical care indicator is measured by documentation of at least 1 CD4 or viral load test performed ≤1 month after diagnosis among persons aged ≥13 years.[[22]](https://paperpile.com/c/nU1tvG/jxCB) This indicator is only available for states with complete laboratory data (at least 95% of laboratory results are reported to the surveillance programs and transmitted to CDC). For the baseline year 2017, linkage to HIV medical care was available for 41 states and Washington, District of Columbia: Alabama, Alaska, California, Colorado, Connecticut, Delaware, Florida, Georgia, Hawaii, Illinois, Indiana, Iowa, Louisiana, Maine, Maryland, Massachusetts, Michigan, Minnesota, Mississippi, Missouri, Montana, Nebraska, New Hampshire, New Mexico, New York, North Carolina, North Dakota, Ohio, Oklahoma, Oregon, Rhode Island, South Carolina, South Dakota, Tennessee, Texas, Utah, Virginia, Washington, West Virginia, Wisconsin, and Wyoming. There are 41 areas with complete lab reporting for 2018 however, the composition of the states changed. In 2018, linkage was available for Nevada but not available for Connecticut. By 2019, the latest year for which data are available, the number had increased to 45.

Linkage to care data by quarter will be calculated for the cumulative number of persons who received an HIV diagnosis by the quarter of interest for the specified calendar year. A 3-month reporting lag is required for calculating linkage to care due to known delays in reporting of laboratory data (e.g., linkage to care through September 2019 is calculated using data reported to CDC’s National HIV Surveillance System through December 2019). Data presented by quarter are preliminary.

*Indicator: HIV Viral Suppression*

HIV viral suppression for a given year was measured for persons aged ≥13 years and living with HIV infection that had been diagnosed by the beginning of the previous year and were alive at the end of the given year.[[30]](https://paperpile.com/c/nU1tvG/o4ap) As an example, viral suppression measures for 2017 was measured among persons whose infection was diagnosed by year-end 2016, who resided in any of the 42 jurisdictions as of their most recent known address during 2017, and who were alive at year-end 2017. The 42 jurisdictions are those that reported complete CD4 and viral load test results to CDC.

HIV viral suppression is available only for states with complete laboratory data (at least 95% of laboratory results are reported to the surveillance programs and transmitted to CDC). The calculation of HIV viral suppression estimates is possible because of improvements in HIV case surveillance and laboratory reporting. For the baseline year 2017, HIV viral suppression was available for the following 41 states and Washington, District of Columbia: Alabama, Alaska, California, Colorado, Connecticut, Delaware, Florida, Georgia, Hawaii, Illinois, Indiana, Iowa, Louisiana, Maine, Maryland, Massachusetts, Michigan, Minnesota, Mississippi, Missouri, Montana, Nebraska, New Hampshire, New Mexico, New York, North Carolina, North Dakota, Ohio, Oklahoma, Oregon, Rhode Island, South Carolina, South Dakota, Tennessee, Texas, Utah, Virginia, Washington, West Virginia, Wisconsin, and Wyoming. There are 41 areas with complete lab reporting for 2018 however, the composition of the states changed. In 2018, linkage was available for Nevada but not available for Connecticut. By 2019, the latest year for which data are available, the number had increased to 45.

*Indicator: PrEP Coverage*

Reported as a percentage, PrEP coverage was calculated as the number of persons aged ≥16 years classified as having been prescribed PrEP divided by the estimated number of persons who had indications for PrEP.[[30]](https://paperpile.com/c/nU1tvG/o4ap) PrEP prescription data values <40 in any jurisdiction are not reported because of reliability concerns. PrEP coverage data for 2018 have been updated using a 2018 denominator for the estimated number of persons with PrEP indications.

Caution should be used when interpreting the PrEP coverage percentages. Different data sources were used for the numerator and denominator; therefore, it is unknown whether all persons prescribed PrEP (numerator) are also contained in the estimate of the number of persons with indications for PrEP (denominator).

The number of persons aged ≥16 years classified as having been prescribed PrEP was calculated using national pharmacy data from the IQVIA™ Real World Data–Longitudinal Prescriptions database. The annual number of persons aged ≥16 years classified as having been prescribed PrEP prescriptions was determined using a validated algorithm that included persons who had at least one tenofovir disoproxil fumarate and emtricitabine (TDF/FTC) prescription for >28 days and for whom TDF/FTC was not prescribed for HIV treatment, hepatitis B treatment, or HIV postexposure prophylaxis. Although IQVIA™ recorded 92% of all prescriptions from retail pharmacies in the United States, prescriptions from closed health care systems that do not make their prescription data available to IQVIA™ (e.g., managed care organizations or military health plans) were not included. Therefore, the calculated values represent minimum estimates of PrEP coverage. PrEP coverage is not available by transmission category due to lack of availability in the IQVIA™ database.

Caution should be used when interpreting PrEP data by race/ethnicity. Race/ethnicity categories available in the IQVIA™ data include White, Black/African American, Hispanic/Latinx, and Other. The number of persons prescribed PrEP for each racial/ethnic group presented was extrapolated by applying the racial/ethnic distribution of known records to those for which data on race/ethnicity were unknown.

To estimate the number of persons prescribed PrEP at the state or county level, a probability-based approach is used to crosswalk between a 3-digit zip code assigned by the U.S. Postal Service and states or counties. Because of reliability concerns, subnational estimates of <40 are not reported.

ACS and U.S. Census Bureau datasets, which include household data on cohabitating same-sex partners, were used to estimate the number of men who have sex with men (MSM) in a jurisdiction. Next, behavioral data from NHANES were used to estimate the proportion of HIV-negative MSM with indications for PrEP. Finally, the number of HIV-negative MSM with indications for PrEP was multiplied by the ratio of percentage of HIV diagnoses (from NHSS) during the specified year attributed to other major transmission risk groups compared to the percentage among MSM in a given state or county. The estimated number of persons with indications for PrEP in the 3 major transmission risk groups (MSM, heterosexuals, persons who inject drugs) in each jurisdiction was then summed to yield a state- or county-specific estimate. State estimates were then summed for a national total of persons with indications for PrEP. For further details, please refer to the report below.

For all states and jurisdictions except Puerto Rico, 2017 data from all sources were used in calculating both the numerator and denominator of the 2017 PrEP coverage estimate. The number of MSM in a jurisdiction is determined using the American Community Survey (ACS) of the US Census, and the ACS is one of the 3 data sources used to estimate the number of persons with indications for PrEP (PrEP coverage denominator). However, prior to 2018, ACS did not include data needed to estimate the number of persons with indications for PrEP in Puerto Rico; consequently, the number of persons with indications for PrEP in Puerto Rico in 2017, was not available. In 2018, the ACS conducted a separate Puerto Rico survey and these data are now available and are being used to determine the number of persons with indications for PrEP in 2018 for Puerto Rico. In addition, 2017 PrEP coverage for Puerto Rico is now provided using the 2018 denominator for Puerto Rico. PrEP coverage for San Juan is not displayed due to reliability concerns.
