# Supplementary figures and images for: America’s HIV Epidemic Analysis Dashboard: Protocol for a Data Resource to Support Ending the HIV Epidemic in the United States
Source: JMIR Public Health Surveill. 2022 Feb 10;8(2):e33522. doi: 10.2196/33522 (PMC8874801; doi:10.2196/33522)

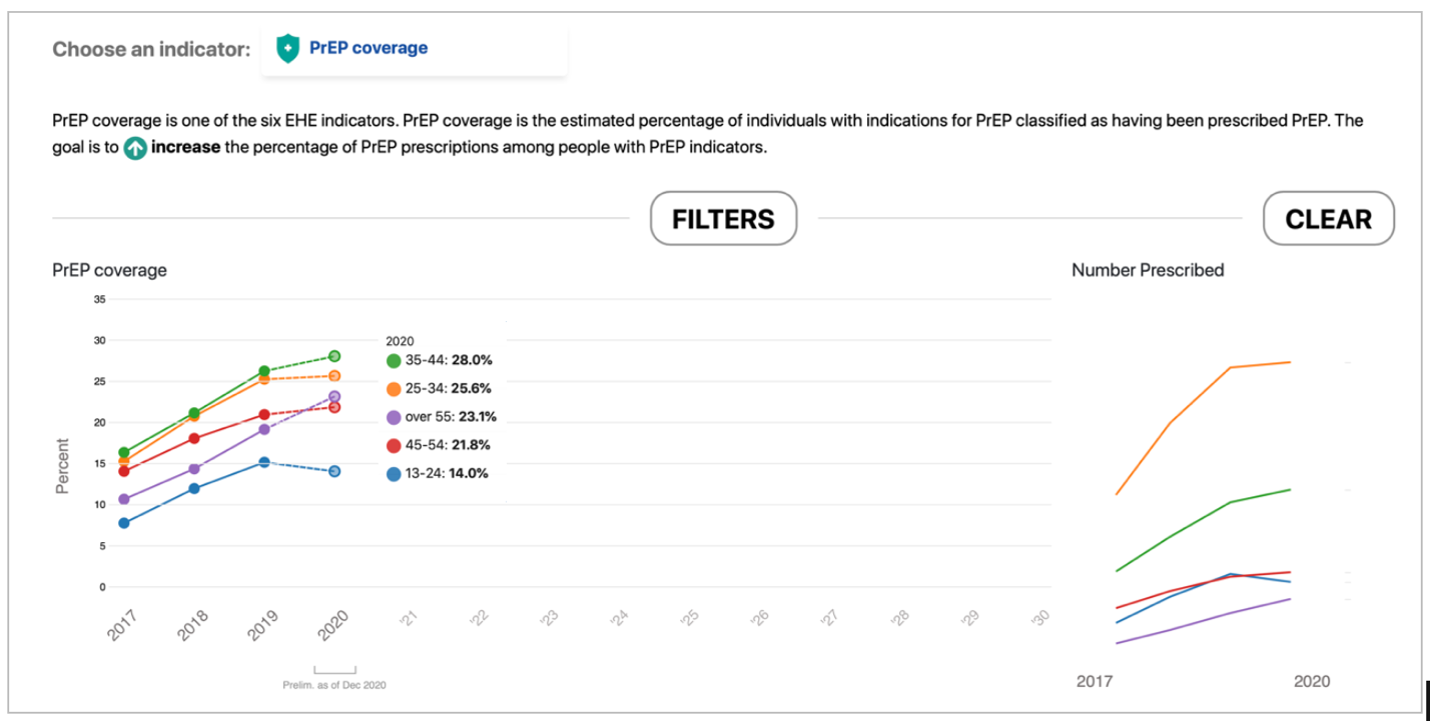

Supplement: Multimedia Appendix 3 [file publichealth_v8i2e33522_app3.png]
